# The Hospital Nursing Supplement

**Published:** 1893-08-12

**Authors:** 


### "The Hospital" Nursing Mirror.

Being the Extra Nursing Supplement of "The Hospital" Newspaper.

[Contributions for this Supplement should be addressed to the Editor, The Hospital, 428, Strand, London, W.C., and should have the word "Nursing" plainly written in left-hand top corner of the envelope.]

### News from the Nursing World.

### THE ROYAL NATIONAL PENSION FUND FOR NURSES.

It will interest many readers to hear that more than 3,000 policies have now been taken out by nurses in this Fund. Its progress has been phenomenal. Without advertising, or canvassing of any kind such as insurance companies resort to, this Fund has steadily grown, whilst its popularity and usefulness have increased. No less than 320 new policies have been issued during the first seven months of the present year as against 257 issued during the same period of 1892. In 1891 an immense fillip was given to the Fund by the reception of the second thousand nurses at Marlborough House, by the President, H.R.H. the Princess of Wales. Very many nurses who were anxious to be present on that occasion flocked in, and the number of new policies for the first seven months of 1891 amounted to 364. We mention this latter fact to show what a boon the Fund has proved to nurses, seeing that 320 new policies have been issued in 1893, without any special attraction, all of which have been taken out spontaneously. This is a proof of the value attached to the Royal National Pension Fund by nurses generally. The invested funds now exceed £150,000.

### COTTAGE NURSES.

This subject is one which commands attention in all parts of the country, and the scheme set forth in The Hospital this week by the Secretary of the Dorset Health Society aims at a new departure. It proposes to apply a certain test to women already engaged in what is termed "going out nursing." The Association proposes that these middle-aged and, probably sometimes, elderly persons should go through a probation of at least three months, and then be subjected to certain examinations to prove their efficiency. Without doubt the plan may prove satisfactory if the women are willing to submit to it. But this is very doubtful, and friction between them and the younger and better trained women is certainly unavoidable. A modified plan has been tried, and is said to have answered well, relations and neighbours of the sick poor having proved willing and eager to accept practical instructions in their homes from trained district nurses. Such teaching is laid down as one of the first duties of the Queen's nurses, who are specially prepared and trained in hygiene and sanitation for the purpose of qualifying them to instruct both by precept and example those with whom they come in contact during their nursing duties. This "health missioning" is assuredly a more practical remedy for village ignorance than the testing or so-called term of training with which these middle-aged persons are asked to comply.

### SILENT SUFFERERS.

"I'm glad he can cry," says the nurse experienced in children and their ways. "His screams are heartrending," says the emotional probationer when she hears the voice of the victim of fire or scalding water. Doctor and nurse have little hope of the poor injured creature who lies cold and silent after a great shock He may reward their care and "come round," but, on the whole, they would rather hear some signs of life. Even the bath, which is now so popular for the alleviation of the first agony of pain, does not immediately subdue the terrors of a scared child. Silent sufferers are the exception, and even amongst adults they are rare enough to be remarkable, except, perhaps, as portrayed in a certain class of literature which deals with an idealised world, in which individuals are all represented "as good as they ought to hav been."

### NURSES AND FIRE DRILL.

The officials at Homerton are now including fire drill amongst the duties of the nurses, and they are wise to do so. It would be well if it were the universal custom in all English schools to teach young people what to do in common emergencies, and to these fire unfortunately contributes a large proportion. It has been inaccurately stated of the movement at Homerton that "The idea of fire drill for women is not new, but this is the first time it has been applied in hospitals, and it is hoped the movement will extend." Homerton, however, cannot claim for its new departure any great novelty, for it only follows the example set it by various general hospitals.

### A KIND SUGGESTION.

The wholesale denunciations against outdoor uniforms for nurses, which seem to be the fashion just now, have somewhat of a feminine ring about them. However, as a pleasant contrast to all this abuse, a country paper gives a letter from a kind-hearted male, who proposes to get up a subscription for providing new cloaks to the district nurses in the neighbourhood. He says they do much good work, and having had personal experience of their constant patience and skilful ministrations, he does not like to see them going about in cloaks which wear and weather have bereft of colour and virtue, and so, instead of proposing to banish this shabby uniform, he suggests that he and other workers of limited means] should club together and see that their friends, the district nurses, have proper cloaks to protect them from wind and rain. This correspondent is a practical man, and evidently believes that "a little help is better than a deal o' pity."

### ADVANCEMENT AT SUNDERLAND.

The training of probationers in the Sunderland Union Infirmary, is indeed a step in the right direction, showing pleasant progress has been made under Miss Cowan's management. Four probationers are now installed for a period of three years' training, and the authorities are to be congratulated on their new departure, which also reflects credit on the able administration of the medical officer.

### TAILOR AND NURSE.

The Poole Board of Guardians has apparently some curious ideas as to the acquirements of a nurse, and also of the remuneration to which such a person is entitled. The Master and the Visiting Committee, having agreed that a nurse was necessary, it was arranged at a recent meeting to advertise for one. Nominally, it is a male nurse for the sick ward who is needed, but the order of "preference to be given to one who knows something about tailoring," rather complicates the matter. Certainly, any man whose knowledge of tailor's work is of the least value, and who is in addition experienced in sick nursing, would hardly think himself adequately remunerated by the £25 per annum, which is all that the Guardians propose to offer.

### AN IMPORTANT SUGGESTION.

It is a true but not new assertion that many people ask advice but very few follow it. Generally speaking, we only want our friends to agree with our own preconceived notions, and when they fail to do this, we think their recommendations are not worth consideration. It was in something of this spirit that the Southampton Guardians, at a recent meeting, received the report of the Visiting Commissioners in Lunacy. The Chairman said :—"On the whole it was of a very satisfactory character," However, when the Commissioner suggested that a night nurse was required, the Deputy President remarked: "He thought the Board equally capable of dealing with the matter, knowing all about it, as an inspector who only came down now and again." If the Board really "know all about it," we sincerely trust that they will now prove also "capable of dealing with the matter," for assuredly it is one on which the Commissioner's advice cannot be ignored.

### EVERYDAY DANGERS.

The late Sister Superintendent of the Watford District Cottage Hospital has been suffering from a very severe illness, "due to the irregular admission of a child suffering from membranous croup." The announcement reads curiously, as the fact of the admission being "irregular" can hardly be said to affect the case. The dangers of diphtheria are well known and can never be lightly esteemed, yet its victims require the best of care, and they are nursed constantly with impunity by both trained and untrained women. The local medical practitioner no doubt regretted sending his patient to the hospital, but probably he had urgent reasons for doing so; at any rate it is hardly fair to saddle an irregular admission with disproportionate blame. The committee have acted kindly and liberally to the ladies who unfortunately contracted the disease, but neither Press nor officials need imagine that the risk of infection is one from which trained nurses can fairly be exempted. To understand accurately where danger lies and how to avoid it is a nurse's first duty. She can, however, no more hope to escape all contact with it than a soldier can trust to his duty keeping him always out of gunshot range.

### AN UNUSUAL COMPARISON.

Surely few qualified nurses can approve the unfortunate simile employed by one of our contributors this week in a suggested comparison between specialists and trained nurses; first-year students and cottagers "who go out nursing." The experienced nurse may acquire a fair amount of scientific knowledge if she has taste for anything beyond the technical teaching given in her training school, but this is a mere drop in the ocean compared with the studies of a doctor, whether consultant, specialist, or general practitioner. Therefore any classification of nurse with specialist is obviously unfair and misleading. And even first-year students will hardly appreciate being bracketted with the unqualified village woman, who must never be confounded with the true nurse. The homely body has a thousand practical good qualities which must be thoroughly appreciated, yet she would certainly never aspire to be ranked with the inexperienced young man of education who is prepared to devote twice as many years as she has given months to prepare for his life work. By setting an inordinate value on the acquirements of trained nurses, their value and distinctive personality are more effectually lowered than they can ever be by kindly shafts of criticism.

### NURSES AT CHICAGO.

The Emergency Hospital which stands in the Exhibition grounds is essentially designed for present use and therefore can hardly be classed amongst the exhibits, although it is, to nurses, one of the most interesting objects at the World's Fair. The bedsteads are of the latest and best patterns, and so are the rest of the fittings in this model temporary hospital. There are many rattan cots, besides the thirty-nine regular beds, and there is an elaborately fitted up operating room. Ten trained nurses are attached to the Emergency Hospital, and they are under the direction of the Lady Superintendent, Miss M. R. Brown.

### ENGLISH AND AMERICAN TRAINING.

Women-nurses frequently ask advice as to their chances of getting work in America, and occasionally a male attendant wants to know whether his services would be appreciated over there. But the time is passed for English nurses of either sex to be in any great demand in a country which aims, in the present day, at any rate, at itself giving as thorough training as can be obtained in any other country. In one department, at any rate, our American friends are ahead of us, for they provide systematic training for male probationers on a plan which England unfortunately seems unable to follow.

### SHORT ITEMS.

Lord Carlingford has devoted £10 a year to the Radstock Sick Nursing Society for the special purpose of supplying blankets to the sick who need them.—The Committee of the Newport (Isle of Wight) District Nursing Society at their recent annual meeting thanked Nurse Butcher cordially for her efficient ministration to the sick poor.—A sacred concert was given on the last Sunday in July in aid of the funds of the Darlington branch of the Q.V.T.I. The Mayor and a number of friends walked in procession from the Town Hall to the public park, where the concert took place. The Board of Guardians have appointed a committee to go into the whole of the nursing arrangements at the Chippenham Workhouse Infirmary.—The Molesey Cottage Hospital Demonstration was held on July 9th, it was organised by the Friendly Societies, and the result does credit to all concerned, for the handsome sum of £72 was collected.

### Unprofessional Criticisms.

By a Nurse Critic.

The critics who write on art, music, sport, or the drama have acquired large practical acquaintance with their special subjects before they begin to find fault, and according to the thoroughness of their knowledge their opinions carry weight with the public. Can details of trained nursing be reasonably criticised by less accurately informed persons?

A qualified nurse, even if her position be only a subordinate one, resents fault-finding from those who do not understand her work and surroundings. Even an experienced doctor, suddenly converted by an accident into a hospital patient, has been known to own frankly that he had never before understood what was really included in the term "Nursing." Lying still in his bed and watching the routine of the twenty-four hours for the first time, it dawns upon him that he is gaining a valuable object lesson, the details of which he had hitherto never even imagined.

In every institution there is room for improvement, otherwise how could there be further progress? for when growth ceases decay surely begins.

A surgeon does not condemn the whole body if a portion of it is less perfect than the rest, and neither does a skilled critic ignorantly pronounce sentence on a whole department because some of its details are incomprehensible to him. Intemperate attacks are invariably made by the unqualified "enquirer," who substitutes discursiveness for special knowledge. These truths are forcibly illustrated by the correspondence in the *Pall Mall Gazette.*

Hospitals and training schools for nurses have a glorious prospect before them, if a recently suggested plan can find supporters. A lady whose knowledge of the actual work in a hospital has been gained by occasional visits to the wards, and from information supplied to her by temporary residents in institutions, has recently made the novel proposal to form a committee of influential ladies and gentlemen to supervise and investigate generally the nursing departments of hospitals. The eligibility of these volunteers is to depend solely on their being subscribers, and the originality of this "qualification for criticism" does credit to the fertile brain of the inventor, although at present the suggestion is somewhat inadequately supported. It has hitherto been conceded that some practical acquaintance with the internal works of an institution was needful to enable any critic to estimate justly its faults and virtues. At any rate, the only evidence to which value attaches itself must come from experts in nursing, on the same principle as governs other fields of skilled labour. For hospital boards, committees, or governors to be expected to submit to the dictation of a self-elected body of "subscribers" is monstrous. The governors and the committees elected by the former certainly have the welfare of each department of their institution at heart, and are unlikely to submit themselves to a little coterie of outside critics.

Most hospitals freely open their doors to that public to whose support their existence is mainly due, and the institutions which adopt a different policy should be advised to abandon it. In the face of this easy access granted to all sorts and conditions of men, it seems somewhat incongruous to talk of making "investigations," as if these had not always been rather invited than shunned. Where closed doors meet visitors whose questions receive no replies then the matter is different for that custom is indefensible.

Miss Florence Nightingale is a marvellous example of far-reaching personal influence, for her opinion carries weight in every quarter of the globe. True, she is an invalid now, but she is also a woman skilled in nursing and sanitation, and up to the present hour she keeps herself in touch with every step of progress in the great nursing movements inaugurated by herself.

Although Mrs. Robert Hunter may be said to resemble the queen of nurses because she too has to bear "a prolonged period of serious ill-health," this alone is scarcely sufficient equipment for anyone aspiring to the position of an expert in nursing matters. It is the practical experience and technical knowledge of Miss Nightingale, and not her invalidism, which gives her an unique position.

Abuse is not argument, and there is little fear that it can permanently influence an English public which has a habit of eventually making up its own opinion, however prevalent the instinct to believe what is "in print."

Let fair and open criticism be welcomed in every department of charitable institutions, but strictures cannot carry weight unless they are uttered by persons skilled in the work of which they aspire to judge.

### Use and Abuse of Uniform.

By a District Nurse.

Many nurses will feel indebted to "An Old-fashioned Nurse" for her article in a recent copy of The Hospital, "Should Nurses be a Distinct Caste?" The subject has been a vexed one for a long time. A district nurse needs a distinctive dress when engaged in her work, but other nurses I fear frequently only adopt an outdoor uniform either from its novelty or (I regret to have to say it) to attract attention. A woman is best dressed when she is suitably clad for the work she has to perform. I have been a district nurse for some years, and I have often been astonished at the arbitrary manner in which I have been requested to wear my uniform. Why should a nurse always be expected to wear one particular style of dress? It is often chosen without any regard to her comfort or appearance. A womanly woman desires to please, and a nurse is a woman, not a nursing machine, and I think it is a duty she owes to her patients and to the general public that while she is attending to her duties she should be neatly, daintily, and becomingly dressed. A fresh cotton gown, short enough to escape the dirt, with large holland apron and sleeves, a neat close-fitting bonnet with clean fresh white strings, and a warm comfortable cloak constitutes my working dress, and when my duties are over for the day I am glad to leave every detail of my "shop life" behind. It is such a relief to leave one's profession for a time, to cease to be the nurse, and to be the ordinary every-day woman. Young nurses are eager to don the distinctive dress, and are proud that the community at large should know they are members of the nursing world. But I greatly question the wisdom of always keeping to one style of dress, one class of thought, and one line of duty. A mechanical woman in a sick room is a great trial; a nurse should always be bright and cheerful, she should endeavour to keep pace with what is passing around her, and the more she can enter into the joys and sorrows of others, the more she will prove that she is the friend and helper of the sick and sorrowful. My ideal nurse is the famous "Miss Florence Nightingale." She did not worry about the silly details that make nurses wish the public would not force unpleasant and unwelcome notice upon them. She was the friend the soldiers loved to welcome, for if she could bring no comfort to the poor weary patient, hold out no hope of recovery or return to his fatherland, still she was a true woman, too large-hearted to leave the sufferer without words of consolation. No one will ever know how wide her influence extended, and will do for all time. Every nurse has opportunities for benefiting the people she works amongst, and I for one do not believe that her duty ends with attention to the actual wants of her people. During the hours she is on duty she should be ready to meet any and every demand made upon her, remembering that she is the servant of the sick. When working hours are over let her forget the dirt, the misery, the sin, and the sorrow that her day has been spent amongst, and let her enjoy the pleasures of life as far as she can.

My people know that when my uniform is absent I will not be troubled with details of cases. Many nurses complain of being dull and people not being considerate. I think this is in a great measure their own faults. People, as a rule, have a horror of disagreeables, and some nurses can talk of nothing but their work. I have been frequently disgusted at hearing topics brought forward that should never be mentioned outside the sick-room. If I want a brief holiday there are always people willing to help me to get it, and if there is a party I wish to go to there is always much interest evinced in my gown. My poor people are quite proud to think I am welcomed by all the people of social standing in the district. I never neglect my duties for any pleasure, and my committee will be willing to bear witness that it is perfectly possible to be actively engaged in nursing work and yet enjoy the ordinary pleasures of life.

About a week ago four women dressed in nurses' cloaks and bonnets (veils and strings) paid our village a visit selling "Wilson's Patent Food" for indigestion, &c. I think all lovers of outdoor uniform would, after seeing these women, have longed, as I did, to make it impossible for anyone except district nurses to wear any such costumes.

### Associations for the Supply of Cottage Nurses.

In a recent number we alluded to the meeting held under the presidency of Lord Brassey to consider the benefit system and the proposed introduction of cottage nurses on the Holt Ockley system. We felt compelled to inquire what amount of training and supervision is considered essential to a cottage nurse in order to ensure that the good work done by them may not in many cases run the risk of being outweighed by their inexperience and want of knowledge. We have received in reply the following communication from the Secretary of the Dorset Health Association, showing how it is proposed to work what is known as the Holt Ockley system in that county under certain modifications:—

"The association is gradually establishing throughout the county district centres in the larger towns, each of them worked by a thoroughly-trained nurse of some years' standing. To each of these centres will be affiliated groups of villages, having, it is hoped, in time their own cottage-nurse living in one village, working within a given radius, having a home of her own, but having where necessary also a lodging to resort to should she be belated in some out-lying village.

In order to supply funds for nurses of both grades throughout the country, the benefit system instituted by Miss Broadwood is being adopted. All members, rich or poor, who pay a yearly subscription according to their means, may claim the nurse at a reduced payment, also on a sliding scale, while non-members pay full price.

By this means the nurses' services are available, without distinction, to all classes of the community, and this, though it will not often be taken advantage of by those who can afford a private nurse to themselves, will, it is hoped, prove a special boon to the lower middle classes, who are unable to afford the expense of a separate nurse, to whom, on the other hand, the system of free nursing is not applicable. The central district nurses will only go out by the hour, the cottage nurses probably either by the hour, the half-day, or the day. Resident nurses by the week are not at present contemplated, as it is found difficult to avoid one member of the club thus obtaining an undue share of service.

In order to provide central district nurses the Dorset Health Association propose to avail themselves of any thoroughly trained nurses. The rules are being made sufficiently elastic to allow of local committees (for the club is essentially an "affiliated" one) to engage nurses from any institution, provided they are both competent and willing to agree to work under the three following restrictions: A, under the benefit system; B, under the guidance of a committee and not of a single individual; C, that the cottage nurses affiliated shall satisfy the requirements of the Central Medical Committee. It will thus be seen that the system differs from that of the Holt Ockley system as hitherto worked,* in that each cottage nurse will be placed distinctly under professional supervision: 1st, that of the trained nurse to whom she is affiliated, and, 2nd, what is still more important, under that of some local medical man, on whose advice and supervision she may count in cases of perplexity, while she must have passed the examination of the Central Medical Committee before being put on the staff at all. In order to provide such cottage nurses it is proposed to make inquiries as far as possible to ascertain what women already "go out nursing" in a given neighbourhood, and to sift all such by careful inquiry, to prove and see who are worth putting on the staff as recognised cottage nurses, and who must be as far as possible dissuaded from undertaking work for which they are not capable. It is only fair that this local investigation should take place before "taking the bread out of their mouths" by bringing in strangers.

It is in order to guide this selection that a Central Medical Committee has been appointed. This brings us to the question of the training of cottage nurses. In Dorset, as in Surrey, a minimum of three months' training (or perhaps "testing" might be a better word) is to be adopted. This is incumbent on all who join the staff as cottage nurses, however experienced and reliable they may be. It is manifest that in the case of such middle-aged women as have gone out nursing for years to the complete satisfaction of a local doctor, this test will be ample to enable them to pass the examination of the Central Medical Committee. Each case will be judged on its respective merits, and according to the place which each proposes to occupy, the inexperienced ones being placed under the immediate supervision of some competent person, and given only such work as an ordinary probationer can be trusted to undertake. The term, "three months' training," has given rise to much misconception, and undoubtedly, if no other test of fitness is required or professional supervision when once launched as a cottage nurse, many unforeseen evils might occur.

Recognising the fact that cottage nurses will merely act as assistants and sub-assistants to regularly trained nurses, many of the said dangers may be removed, and an existing body of local women organised and utilised. That any idea of their being mistaken for or entering into competition with hospital-trained nurses should be as impossible as for a medical student in his first year to be mistaken for a specialist. Should any of them aspire to rise to the higher grades of the profession, they would, of course, have to enter through the usual channels and to go through a prolonged course of hospital work.

With regard to this proposed training, it is suggested to arrange for it in one of the following ways: Either by a course of three months in a county or cottage hospital, or in the case of a local midwife, for six weeks or three months' training or testing under a certified London district midwife, or at a maternity home, or by a course of district work under some of the central district nurses. In order to raise money for the expense of such training a small fund has already been raised in the county, and it is hoped that more will be forthcoming, while most of the hospitals have agreed to assist in the training and testing.

We learn both the County Councils of Shropshire and Oxfordshire have set apart a certain portion of technical money for the training of local nurses—in maternity and general nursing—but upon what lines we are not yet aware, and it is hoped that sooner or later other county councils may see their way to some provision of the sort. We shall watch with interest the result of the experiment about to be worked out in Dorset, if it succeeds, it may encourage others to essay some similar system in providing nurses for the sick in remote country villages, while at the same time surrounding the experiment with reasonable safeguards."

* This plan it is proposed to work in Surrey.

### Male and Female Nurses in German Catholic Orders.

By Dr. Köllen, Sanitätsrath, St. Hedwigs Hospital, Berlin.

The number of religious orders and similar associations of the Catholic Church attending to sick nursing in Germany is very large; in Prussia alone there are 49 different orders with 886 stations, and amongst these there are 5 male orders with 34 stations and 517 members, and 44 female orders with 852 stations and 7,352 members.

The Catholic Church predominates in most of the German States over the Protestant, unlike Prussia; an idea may be formed from these figures of the great extent of nursing by religious institutions.

Amongst the most widely spread and active orders of Prussia, as also of the other German States, there may be especially mentioned the Alexian Brothers, the Brothers of Mercy, the Franciscans, the Boromaeous, the Poor Maids of Christ, the Sisters of Mercy, the Elisabethians, and the Gray Sisters. The cause of this manifold form of religious nursing orders must, however, not be sought in their various objects and regulations, since these are on the whole the same, and corresponding to the common spirit that pervades these institutions. Although their activity presents a varied appearance, the cause of this must be sought for in the depth of the religious life of the Catholic Church, a depth that permits of placing in the foreground in various orders one and another side of merciful activity. Most of the religious orders devote themselves to the nursing of the sick in hospitals; some confine themselves to district nursing, while others again do both; a few follow certain special lines of nursing, as the Alexian Brothers, for example, who devote themselves to the care of the insane. Notwithstanding these differences, the training is everywhere inspired by the same points in view, and the same general rules, and the description that is to follow may hold good in general for all orders now existing in Germany, and all other European countries, allowing of course for some slight deviations resulting from the national character of the people, and the degree of civilization.

The age of the applicant to one of the nursing orders must not exceed thirty years. Exceptions to this rule can only be made upon the special permission of the higher religious officers. The applicant must furthermore furnish the superior of the order with a certificate from the worldly, as well as spiritual, magistrate of his home, testifying to the good character of the family, and his own faultless life. Should he have been a soldier, an attest must also be provided by the military authorities as to his conduct during the time of service. The applicant must be in good health and free from any bodily defect. His temperament must be cheerful and excluding any tendency toward melancholia and conscientious scruples, and be manifested by a friendly appearance. On the other hand, his admission is dependent upon the general impression of his entire mental development, as also of his character, and upon his being guided by the love of God and mankind, and filled by the desire to alleviate suffering by his general bearing as well as by his nursing.

As to his mental education, a sufficient knowledge of all elementary instruction given in school is at least presupposed, and a certain degree of intelligence. The superiors of the orders must feel convinced that the applicant has previously tested himself thoroughly and earnestly, *i.e.*, that he has remained faithful to his plans for at least one or two years, and that he has prepared himself by means of good deeds and renunciations for this profession. Upon admission the candidate is handed over to the mother institution with which a larger hospital or asylum is connected, and here the first instruction is provided in monastic life and the nursing of the sick. To this end daily instructions are given of a theoretical as well as practical character. This is at first confined to all kinds of domestic work, such as washing, ironing, cooking, the cleaning and airing of rooms, then to the taking hold of, the lifting and carrying of patients, the cleansing of wounds, the disinfecting of instruments and of the operating-room, and the application of bandages, poultices, leeches, cupping, the administration of hypodermic injections, &c.

This instruction is furnished by members of the orders who have already obtained a sufficient degree of experience, and, in the more important branches, by the physicians themselves. In the course of this first instruction the superiors also obtain an idea of any special adaptability and inclination on the part of their pupils. Some manifest an especial talent for cooking, others for washing or needlework, and still others for various kinds of handicraft. Many wish to be occupied with true nursing, and amongst these some are especially adapted to the nursing of medical, others of surgical cases, and of children and the aged. Small, however, is the number of those who assist directly at operations, and those who pass the apothecary's examination and are able to take in hand the druggist's department of a hospital.

After an education at the mother institution, which extends over one and one and a half years, the candidates pledge themselves for four, and in some religious orders for five years. They are then sent away from the mother institution as novices to daughter institutions, *i.e.* to other hospitals or insane asylums belonging to the order so as to perfect themselves by continuous daily practice.

Here their education becomes further developed in all branches, alternately in the medical, surgical, and children's wards, in the more severe cases, the lighter and the chronic, or on the other hand in the kitchen or the drug store, always, however, under the supervision of superior and experienced officers.

During this time those who are especially talented in assisting at operations, or in the apothecary's department receive a special education under the guidance of the resident physicians or the apothecary respectively. For all, however, in addition to the technical training in sick nursing, the most conscientious and faithful obedience to the physicians' orders, and especially amiable and loving treatment of the sick, is regarded as the great end to be attained. After this period of training has also been completed, and after the novice has shown himself competent, he returns once more for a year to the mother institution, in order to complete his education under the supervision of the superior general, to obtain the finishing touches, so to speak, but especially to test himself once more before taking the eternal vow. Up to this time the novice is at liberty to leave the order; if, however, he does not wish to do so after a final self-examination, and if he is deemed worthy by his superiors, the taking of the eternal vow before the bishop takes place, and his formal admission to the order. Herewith the novitiate is ended, and the formally-accepted is now either retained in the mother institution or sent to some other house belonging to the order, where he is placed in a position where he is upon his own responsibility.

This is the course of training, with slight deviations in all orders, devoted to sick nursing in Germany.

That such an education is a proper one is demonstrated by the results reached, especially within late years, in the nursing of the sick by Catholic orders.

Although State and community have bestowed their attention and care upon sick nursing more and more, it remains an undisputable fact that the hospitals of these orders not only maintain their position, but daily grow in importance, and in the esteem and confidence of all classes of the population.

The soil upon which these fruits have ripened, and from which the members of the orders, even after their training, draw strength and enthusiasm, is the strict and uniform care of the spiritual life and that complete devotion to suffering humanity, from the love of God, based upon the evangelical rules of the Catholic creed.

### Everybody's Opinion.

*[Correspondence on all subjects is invited, but we cannot in any way be responsible for the opinions expressed by our correspondents. No communications can be entertained if the name and address of the correspondent is not given, or unless one side of the paper only be written on.]*

### THE LONDON HOSPITAL.

By "A Constant Reader."

Most nurses must appreciate the position taken by The Hospital with regard to the London. The fact that patients, nurses, servants, &c., form a kind of family composed of over a thousand members accounts for a few grumblers being always present. Even in a private household consisting of, say, a dozen persons, somebody occasionally indulges in fault-finding, and outsiders do not generally admire or encourage the tendency. I could wish that qualified nurses, or competent sisters, might take courage and speak out plainly, after the fashion of The Hospital. I have been a frequenter of the London, amongst other hospitals, for many years past, and have visited its wards at all hours alone, as well as with the much-abused officials, and I can truly say that the institution can compare favourably with any other in London in comfort, kindliness, courtesy, and contentment.

By "A Scotch Nurse."

Will you kindly allow me as a certificated nurse of seven years' standing to express the opinion of myself and many other trained nurses on the recent attack made by the *Pall Mall Gazette* upon the London Hospital. I do not know whether to be the more astonished at the consummate conceit and ignorance of the "Special Commissioner," or at her strangely-perverted ideas of honour in thus publicly attacking a great hospital of which she has such superficial knowledge. She became an inmate of it with no intention, it seems, of doing good work, but in the unenviable position of a "spy." I am sure that any trained nurse who has the cause of her profession at heart must look upon such conduct with contempt. She states that she entered the hospital for "three months' training," during which time she "took a vacation," and yet she considers herself qualified to judge, to condemn, and to suggest improvements, although admitting that she does not understand the system of management. She states that there is an "undercurrent of grumbling which the nurses dare not openly express, lest they should be discharged, as those who have ventured to complain in the past have summarily been"; we also hear that "not one in twenty of the nurses who come to the hospital remain for any length of time." How is it that complaints are not heard from the nurses who depart in this manner? Having left the hospital they could make their statements with impunity. I do not myself know the London Hospital, but I have met nurses who have belonged to it, and from them I have heard no complaints. It is noticeable that attacks of this kind are usually made by untrained probationers, who know nothing of real hospital work. Of course every nurse owns that the life is a very laborious one. It must necessarily be so, yet a probationer always enters for at least a month on trial, and has every opportunity of judging of her own capacity for the work before deciding to remain. Having so decided, it is unreasonable to grumble at that which is undertaken voluntarily. With regard to the complaint that all the opinions of the Matron regarding the character and work of the nurses must be formed upon the reports of the sisters, I would only say that, although Miss Lückes is well known as being one of the most efficient matrons of the day, yet it must be impossible for her to have 'some sixty wards,' containing perhaps 700 or 800 patients, under her own personal daily supervision. Therefore who is so able to report upon the work of the nurses as the sisters under whom they are trained? It is to be hoped that such ignorant and foolish attacks may not be repeated, for they are not made by any true friend to the nurses.

### SHOULD NURSES BE A DISTINCT CASTE?

"Another Nurse" writes: I read with great pleasure what "An Old Fashioned Nurse" writes on the question. "Should Nurses be a Distinct Caste." She has said exactly what needed saying, and it is to be hoped that matrons or superintendents will set their faces against the prevailing out-door uniform for nurses. If quiet effect and convenience and warmth and coolnes can be met in one costume by all means let us have it; but at present the cloak, bonnet, and veil are exceedingly harassing in rain or wind, and are about as inconvenient a combination as one can imagine. Although "riding, boating, and tennis" have to be given up to a great extent when nursing is to be the life-work, the nurse or probationer who carrries with her to her hospital the healthy liking for out-door amusements might be able to get an occasional set of tennis with friends were she not doomed to go to the house clad in the inevitable cloak and bonnet. Who could play comfortably on a sunny court in a shadeless bonnet; or who could do anything on a windy day either at tennis or boating if obliged to wear anything so insecure as the general run of bonnets, handicapped furthermore by a yard or two of flapping veil? "The one thing for which it is fitted is to attract attention in the streets"—this again is a very truthful remark. From my own experience and the experience of others, I know that although as a rule safe from active impertinence, never have I been so much annoyed by the stolid "British stare" as when I wore the honourable, but remarkably unsuitable uniform of a nurse.

### DISTRICT NURSES.

Miss Louisa Twining writes: I should like to direct attention to two important statements on following pages in a recent number of The Hospital, for both cannot be true, and I leave your readers to decide which opinion they will endorse. On page cliv. we read that Miss Hampton, of Baltimore, states in a paper just read and published, "In district nursing we are confronted with conditions which require the highest order of work, but the actual nursing of the patient is the least part of what her work and influence should be among the class the nurse will meet with. To this branch of nursing no more appropriate name can be given than 'instructive nursing,' for instructive in the best sense of the word it should be." On page clv. we read that "while all the nurses for cottages had received some training, skilled hospital nurses were not engaged" (two reasons being given). "It was also found that as a rule a highly skilled nurse was not needed for the majority of cases of illness in the villages." The period of training given is for three or six months. It is surprising that this should be considered sufficient for those who in this work must be left so largely to their own resources and responsibility. Again, surely we are not to make a distinction as to qualifications for nurses employed "amongst patients of a higher order in the social scale." The lower the class the more does it need the tact, experience, and training of a higher intelligence.

*** The "statements" referred to are simply expressions of opinion by a correspondent, and so found a place in our columns.—Ed. *T. H.*

### Midwives' Registration.

The Midwives' Registration Committee brought their labours to a conclusion on Tuesday. The report recommends that a system of examination and registration of midwives should be established, and that, for the purpose of admission and examination of women desiring to act as midwives, the General Council shall be invited to frame rules for regulating—(1) The admission to the register either by (*a*) practice, or (*b*) examination; (2) the conditions of admission to such examinations; and (3) the conditions of such examinations. These rules and regulations should, in the opinion of the Committee, be subject to confirmation by the Privy Council, and in the event of the General Council failing to make such rules as the Privy Council can confirm, the committee recommend that the Privy Council should invite some other medical body or forthwith cause the proposed rules and regulations to be framed for the purposes required, and that such rules shall take effect as if they had been made by the General Council, and confirmed by the Privy Council. The committee also recommend that the duty of carrying out locally the provisions of the Act that will be required should be placed in the hands of the county councils. They also are of opinion that greater facilities for the study of midwifery should be provided in workhouse and lying-in hospitals.

### Appointments.

[It is requested that successful candidates will send a copy of their applications and testimonials, with date of election, to The Editor, The Lodge, Porchester Square, W.]

Liverpool City Hospital, Netherfield Road North. —Miss Jessie Main has been appointed Matron of this hospital. Miss Main was trained at St. Thomas's Hospital. She was assistant to the Lady Superintendent at the Parish Infirmary, Liverpool, and had temporary charge of infectious block, St. Thomas's Hospital, and of the out-patient department at Charing Cross Hospital. We wish her success in her new work.

West Ham Hospital.—Miss Ethel Willmott has been appointed Matron of the West Ham Hospital. Miss Willmott trained as lady pupil at the Boston Hospital, Lincolnshire, and has for some time been Sister of Wards at the Devon and Exeter Hospital.

Clapham, Brixton, and Surrey Association of Trained Nurses.—Miss Annie Cheatle, for five years Matron of Torbay Hospital and Matron of Paignton Hospital, has been appointed Lady Superintendent of the Association of Trained Nurses, 210, Clapham Road.

Miss H. Lawrence, recently appointed to Longton Cottage Hospital, was Matron of the Dorset County Hospital, Dorchester. She tells us there is not a Cottage Hospital.

### Notes and Queries.

### Queries.

(166) *Male Nurse.*—Can anyone tell me where male probationers are received for training?—*H. L.*

(167) *Dispensing.*—How can a district nurse become a dispenser?—*Sandy.*

(168) *Infirmary.*—Will three years' training in a good workhouse infirmary count the same as in a general hospital?—*Anxiety.*

(169) *Ambulance.*—Will St. John's Ambulance Nursing Certificate be of any use in helping me to a post as probationer?—*Enquirer.*

(170) *District Nursing.*—Where can I obtain proper books for district nurses' reports?—*Llandudno.*

### Answers.

(166) *Male Nurse (H. L.).*—Read an article on this subject in The Hospital of October 29th, 1892.

(167) *Dispensing (Sandy).*—In Burdett's "Hospital Annual" (Scientific Press, 428, Strand, price 5s.) there is a chapter on Dispensing, which will tell you everything you want to know.

(168) *Infirmary (Anxiety).*—If you go to an infirmary where the Matron and Charge Nurses are fully trained you will get similar instructions to that given at any general hospital. Your question is not quite clear. A probationer after complete infirmary training would probably be eventually advanced to the post of Charge Nurse or Sister in the same institution if she proved suitable.

(169) *Ambulance (Enquirer).*—Not in a hospital where you enter for a complete course of training; in a cottage hospital or home it might be deemed an advantage. The Ambulance course is useful for every home emergency.

(170) *District Nursing (Llandudno).*—You can get the books from Lowes, 157, High Holborn. This answer was given to query 53.

### For Reading to the Sick.

### THE BEST BED-MAKER.

A good night's rest in a comfortable bed is one of the best preservatives of health, as it recruits our weakness and restores strength to the worn flesh. When we are young and hearty, we are not very particular as to how our bed is made —that we have one to go to is sufficient for us, be it hard or soft, short or long, it makes little difference to our repose. We lie down, and in a few minutes drop into forgetfulness of the pleasures or petty annoyances of the past day, rising up again "like a giant renewed in his strength" to run our next twenty-four hours' course. But when our youth is gone or illness assails us things are at once changed. Thoughts of past sorrow or prognostications of future difficulties and disasters keep us awake, we turn and tumble about, grumble at the bed, and ultimately at the bed-maker. The bed is too hard or too soft, we say; there are lumps which hurt us. Why are the clothes so thick and tucked in so tightly? or, on the other hand, why are they so thin and so loosely arranged? We shake up the pillows and wrestle with the bolster, but all is unavailing, and we cry out in our discomfort and misery, "Would God that it were morning!"

There is no doubt that there are some very bad bed-makers, careless, slovenly servants who do not strip and air the beds and turn the mattresses every day, but just huddle on the sheets and blankets anyhow, converting the coverlet into a coverslut to save time and trouble. With much satisfaction human nature will lay the blame on these idle, profitless people, but shall we not look at home and see how we personally have helped to set thorns in our pillows; for morally some of us have indeed been very bad bed-makers.

It is proverbial that everything appears in the darkest hues to those who are sleepless in the dead of night, how then will it be if we have been careless, slovenly servants of our Master? If we have wasted the goods He has given us in riotous living, and been slovenly in our prayers or neglected them altogether? Under such circumstances we cannot expect to lie quiet and content, for our consciences will remind us of our shortcomings, and regrets for wasted opportunities will prick our hearts as the quills of a bad feather bed annoys our bodies.

Prayer, patience, and a conscience at peace with all men will smooth our couch and draw us nearer to Him who has promised to "make all our bed in our sickness." We cannot do better than hear the words of Thomas Fuller on this subject. That wise and quaint old divine says, "When a good man is ill at ease God promiseth to make all his bed in his sickness—pillow, bolster, head, feet, sides, all his bed. Surely that God who made him knows so well his measure and temper as to make his bed to please him. Herein His art is excellent, not fitting the bed to the person, but the person to the bed, infusing patience into him." Then we will pray, in the words of the dear old Evening Hymn:—

"If in the night I sleepless lie,
My soul with holy thoughts supply;
Let no ill dreams disturb my rest,
No powers of darkness me molest."

### Novelties in Household Linen.

The household linen supplied by Messrs. Robinson and Cleaver, of Belfast, to H.R.H. the Duke of York for the furnishing of York House, comprised some very charming things. One excellent detail in the designs of the tablecloths ought to be universally adopted. Everybody knows how many artistic borders are wasted because from their position they are invisible to the guests. Messrs. Robinson and Cleaver have placed their beautifully designed wreaths in a wiser way, for they are always seen upon the table, and not hidden at all. This firm has long been known to our readers for their excellent collars, cuffs, and aprons, and many are doubtless aware that capital underclothes are also supplied at moderate prices and of any pattern specially desired by customers.

### The Book World for Women and Nurses.

[We invite Correspondence, Criticism, Enquiries, and Notes on Books likely to interest Women and Nurses. Address, Editor, The Hospital (Nurses' Book World), 428, Strand, W.C.]

Nursing: Its Principles and Practice. For Hospital and Private Use. By Isabel Adams Hampton. (Philadelphia: W. B. Saunders. 1893.)

Under the above title we welcome what we regard as a very valuable addition to the books already written on the important subject of nursing. Indeed, it is not too much to say that in no single volume yet published has the subject been so scientifically, carefully, and completely treated as it has been by Miss Hampton in the book now under consideration. Whether as a teaching manual or a book of reference, the thoroughly practical instruction which it conveys is sure to obtain for it a wide circulation.

The course of study prescribed is divided into junior and senior class work, each division occupying a year. Anatomy and practical nursing are the subjects given for the first year, and the last is made to include a tolerably comprehensive study of materia medica. Curiously enough, while chloral mercury, nux vomica, even strophanthus and veratum vivide, receive special notice as drugs presumably in more or less common use, quinine, and the modern antipyretics, antipyrin, and antifibrin are omitted. The teaching of the second year, to use Miss Hampton's own words, "refers more particularly to the changes which take place in the systems of the body when invaded by disease, to the signs of these, and the duties of the nurse in regard to them." For both years of the course a list of lectures, delivered presumably by the physicians and surgeons connected with the special hospital, is given separately. These lectures, covering a wide range of subjects, are made as far as possible to correspond with the teaching work done in the classes.

In Chapter II. we are introduced to the hospital ward, and detailed information is given regarding its discipline, not forgetting the general application of the rules laid down to nursing done in private houses; thus, when a room opening out of the hospital ward is spoken of as a ward office, and a place which should contain the medicine closet, the reader is also reminded that in a private house a room adjoining the sick-room should be set apart for the reception of everything in the way of utensils, medicine, &c., as the sight of such things are likely to be disagreeable to the patient. The average free ward is calculated to contain from 25 to 30 beds, and good practical advice is given as to its ventilation, furnishing, &c. The work of such a ward is done by four nurses, whose duties are carefully detailed. The temperature nurse does the work which her name indicates; two nurses attend to the wants of the patients generally, one on the right side and the other on the left side of the ward; the fourth nurse, called the probationer, or junior nurse, assists in bed making, the listing of soiled linen, &c.

In the last half of this chapter, hospital etiquette is discussed, and much valuable advice is given on this subject to the nursing staff; we take a few examples: "The nurse should never forget to be particularly attentive and kind to a new patient; the dread of entering a hospital is bad enough, but much of the gloom can be removed by the bright, cheerful greeting, &c., &c." "An *esprit de corps* should prevail among the nurses. From the moment that personal jealousy, discord, and fault-finding appear the standard of the work is lowered. When strangers come to visit the hospital, and through some neglect at the door appear in the ward unattended, the nurse should go at once to such, ask if there is anything she can do for them to spare them the extremely uncomfortable sensation of having unpardonably intruded." The writer of the review has himself had practical experience of such a position in the case of hospital governors, who not being themselves doctors are therefore unaccustomed to visit hospital wards, have been hindered in doing their duty by the discomfort which they experienced in making such visits when not properly attended. "An intimate friendship between the head nurse and the pupil ought never to exist." We might multiply these examples, but enough has been written to show the kind of advice given and the practical experience of the author.

The instructions contained in Chapters III., IV., V., and VI. on ward supplies, bed-making, temperature taking, and hygiene generally are all sound, and practical. The advice to nurses to be economical without being parsimonious in dealing with food, surgical dressings, &c., is needed, and is enforced by the observation that by care exercised in this way, "the nurse may herself become a liberal contributor to charity, enabling the hospital to provide for two patients, when otherwise only one could be supported." A useful nurse's toilet basket is vescribed in Chapter IV., figured as a frontispiece to the book on Plate I. Plate II. shows a surgical operation-bed, which is elsewhere fully described. We quite agree with Miss Hampton that with regard to the coverings of beds generally, the usual heavy white counterpanes should be condemned. Bed sores are well treated of at page 87, *et seq.*, but we should scarcely have mentioned the tips of the ears as "among the most common sites" for these lesions, ner perhaps have advised iodoform (page 115) as a first application to a superficial abrasion, or to reddened skin before an abrasion had actually appeared. Boracic ointment, or a little boracic acid in fine powder, would surely answer the purpose in such slight cases, and the very objectionable smell of iodoform be avoided, a matter worthy of consideration, at least in private houses. The retention of these dressings by the application of "celloidin solution" is a good suggestion; whatever they consist of, the important matter is to keep them in position. On the subject of ventilation the reader of the book will find much good practical advice and suggestions, which are not limited in their application to the hospital world. Chapter VI concludes with that part of the care of the dead which specially concerns the nurse. The provision advised to be made against the escape of post-mortem discharges is too often neglected, and in private houses should always receive attention. In the chapter on baths, which is otherwise very complete, no mention is made of the excellent plan for reducing temperature by cold effusion, devised by Thomas of New York, and figured in his book In Diseases of Women. Thomas's plan, in which the water-proof trough is fastened below a bamboo couch, has, we know, been found very serviceable in the East. Its simple construction, and easy management recommends it as a very useful addition to the resources of a physician engaged in private practice, and unable to obtain the special appliances and trained assistance provided for him in hospitals. The chapter on the preparation of the various solutions of carbolic acid and perchloride of mercury used for disinfection and in the practice of antiseptic surgery, is well and cleverly written, but since Miss Hampton's book is intended as a guide to practice both in hospitals and private houses, perhaps a single paragraph devoted to a few simpler statements regarding these matters might have been added with advantage. In a private house a nurse rarely has measuring glasses and weighing machines at her disposal, and it is useful for her to know, for example, that an ordinary sherry glass moderately filled holds about two ounces; a wine bottle (quart), filled up to the foot of the neck, holds twenty-four ounces; and that, therefore, half a sherry glass of pure carbolic acid added to one wine bottleful of water, and well shaken up,

**THE BOOK WORLD FOR WOMEN AND NURSES**—*continued.*

will give a 5 p.c. solution sufficiently exactly for ordinary purposes of disinfection. While a saturated solution of boracic acid, an excellent gargle, can be at once prepared by adding the crystals to water in sufficient quantity to leave a sediment at the bottom of the vessel—the approximate measures on page 225 are scarcely correctly given.

In England, two desert spoons, not two tablespoons, must nearly contain a fluid ounce; an ordinary wine glass (sherry) holds a little over two ounces, not one and a half ounces as stated. Still keeping the full purpose of Miss Hampton's book in view, we might suggest an addition to the advice given about vaginal douches at page 163. In private practice these douches are conveniently administered with the patient lying on her back on the side of the bed, her feet resting on two chairs. An indiarubber sheet laid under the patient conducts the water into a foot pail, the whole operation being conducted under a suitable light covering by the nurse. Similarly the directions given regarding the preparation of hot fomentations at page 192 might be supplemented by pointing out that an excellent substitute for the "Stupe-wringer" of the hospital will be found in a common bed-room towel, in which the flannel may be placed and wrung out over an empty wash-hand basin. The instructions as to the use of the catheter and the administration of enemata are well and clearly given. Subcutaneous remedies and their administration receive full attention at page 213, *et seq.*; the antiseptic precaution to be observed are very correctly detailed, though we cannot, perhaps, fully endorse the advice given as to keeping the syringe and needle, even of an instrument in constant use, in a 1 to 20 solution of carbolic acid. A subcutaneous syringe cleaned before and after use with the carbolic solution, and afterwards with absolute alcohol, receives, in our opinion, sufficient attention; the needle if left in a carbolic solution is very rapidly destroyed. It is a noticeable feature in this book that a short but distinctly scientific explanation of the object aimed at is always associated with the special directions given for the practical work to be done, the intelligent reader being greatly assisted in this way to master details which would otherwise easily be forgotten. Thus, the chapter which deals with disinfection opens with a short dissertation on the subject of bacteriology and the important part which micro-organisms play in the history of inflammation and infectious diseases; while the directions given regarding the making of poultices, the application of liniments are prefaced by a short account of the action of these remedies, so far as it is yet known. These explanations are all clearly given, and, so far as they go, are quite up to date. Surgical nursing occupies eighty pages of Miss Hampton's book, and begins with a more extended reference to the facts of bacteriology and the life history of these organisms; asepsis and antisepsis are defined, and full instruction as to the nurse's practical work follows. In the preparation of the area to be incised by the surgeon, we cannot express approval of "a green soap dressing applied, and left on for at least two hours"; but suppose that this must have been the usual practice of the surgeons under whom Miss Hampton worked, thorough cleaning with soap and water is, of course, necessary, but as a dressing to complete the purification we prefer a 1 to 40 solution of carbolic acid, to be retained for several hours, and usually until the patient is placed on the operating table. A good dressing carriage, very fully equipped, is figured on Plate VII. An Arnold's steriliser, elsewhere recommended for this purpose, and figured on page 149, contains the sterilised dressings. Gynæcological work, the operating room with its preparation by the nurse, and hæmorrhage are all discussed in separate chapters, and these are among the best in the book. The antiseptic precautions observed or provided for are very elaborate, and are all carefully detailed. Chapters XIX. and XX. contain the kind of information regarding bandaging, accidents, medical emer-

### THE BOOK WORLD FOR WOMEN AND NURSES—*continued.*

gencies, and poisoning, as it is usually given in books on "First-Aid," and scarcely calls for much notice here. We are disappointed, however, to find that the application of starch bandages, which we regard as perhaps the most useful of all the fixed bandages, is scarcely more than mentioned (under the head of crinoline bandages) by Miss Hampton. Starch bandages strengthened by pasteboard, and in the treatment of fractures in England associated with the name of Mr. Sampson Gamgee, F.R.S.Edin., deserve more attention. In the treatment of sprains, nothing is said regarding the remarkable results to be obtained by firm bandaging over a mass of cotton wool. The few appliances needed in medical nursing are given on page 317, *et seq.* The chapter on diet and the feeding of patients is good. The necessity that there is for the nurse to acquire some sound practical knowledge regarding the preparation of invalid dishes in the kitchen under a competent instructor is insisted upon, and the reader spared the usual receipts for the manufacture of beef tea and calf's-foot jelly.

The peculiar duties which this monthly nurse is called on to discharge during and after labour, with the management of the infant in the early months, are separately treated of in Chapters XXIV. and XXV. Here, again, there is much to be said in praise of the detailed instructions given regarding the antiseptic precautions to be observed in midwifery. The nurse is apparently encouraged by Miss Hampton to observe the progress of the labour from time to time by examining the patient, such a sentence distinctly advises it. "When the external os is three inches in diameter, it is time for the membranes to rupture, and in a primipara this is the time to send for the physician"; but in private practice at least this can only rarely be permitted, and the nurse, unless in cases of emergency, should always obtain the permission of the medical attendant before she attempts it. In private practice a small bowl—a soapdish answers the purpose very well—will greatly assist in protecting the bed, or used as a scoop in removing blood, &c., and will enable the nurse to dispense with the more elaborate pads and cushions described at page 368. Very little is said by Miss Hampton regarding the evacuation of the bowels and bladder of the mother after labour. For many years past we have been in the habit of insisting that these matters should be arranged for in the usual way on a commode placed close to the bed. The fear of hæmorrhage in all ordinary cases after the first twelve hours is, we believe, quite unfounded. When the patient is allowed to assume the sitting position the catheter is rarely needed, the emptying of the bowl is greatly facilitated; last, but by no means least, the blood clots, &c., which under other circumstances would remain ponded up in the vagina, gravitate easily into the receiving vessel. For ordinary purposes the salicylic cotton taken from a fresh packet makes a safe and convenient pad to place over the vulva; "a sterilised napkin made of absorbent cotton and gauze" is not easily carried about, and is too often rendered useless by the handling that it receives in the sick room. The frequent changing, at first every two hours, of these pads, must be attended to carefully by the nurse. Miss Hampton's book concludes with a chapter of Notes on some Medical Diseases; these are short, and, so far as they go, accurate; they do not need special notice here.

We have submitted Miss Hampton's book to an unusually careful analysis; and we have done this because we believe that it well deserves such treatment at our hands, but also because in our opinion it supplies a distinct want in the literature of the subject as that is at present represented by the books published in England. The life of the patient in not a few cases depends more on the care of the nurse than on the advice given by the physician; in many cases the work of the physician or the surgeon can only be efficiently accomplished when it is intelligently seconded by the nurse; thus the thanks of both doctors and nurses are due to Miss Hampton, who by her work and her book has done, and is still doing, so much to raise the standard and extend the field of the nurses' training.

